# The Changes in Cross-Resistance, Fitness, and Feeding Behavior in *Aphis gossypii* as Their Resistance to Sulfoxaflor Declines

**DOI:** 10.3390/insects15120920

**Published:** 2024-11-25

**Authors:** Mingyuan Lv, Wei Wang, Fengyun Fang, Xiaowei Fu, Gemei Liang

**Affiliations:** 1 State Key Laboratory for Biology of Plant Diseases and Insect Pests, Institute of Plant Protection, Chinese Academy of Agricultural Sciences, Beijing 100193, China; 13963186405@163.com (M.L.); ffy918636@163.com (F.F.); 2 Department of Plant Protection, Henan Institute of Sciences and Technology, Xinxiang 453003, China; 3 Key Laboratory of Integrated Pest Management on Crop in Northwestern Oasis, Ministry of Agriculture and Rural Affairs, Institute of Plant Protection, Xinjiang Academy of Agricultural Sciences, Urumqi 830091, China; wlzforever2004@sina.com

**Keywords:** *Aphis gossypii*, sulfoxaflor, resistance-decline, EPGs, fitness

## Abstract

The increasing resistance in *Aphis gossypii* field populations to sulfoxaflor and many different types of insecticides represents a significant challenge in protecting cotton production in China. Although resistant pests were able to regain their susceptibility to insecticides after the reduction in insecticide applications, some of their biological parameters remained different from susceptible strains. The resistance to sulfoxaflor was unstable in *A. gossypii* after the loss of selective pressure. The strain with declined resistance (Sul-D) (RR = 1.11-fold) restored its susceptibility to sulfoxaflor, acetamiprid, and imidacloprid after the sulfoxaflor-resistant (Sul-R) (RR = 51.57-fold) was maintained without insecticide pressure for 22 generations. Sul-R had a relative fitness of 0.87, and the Sul-D strain still had a relative fitness of 0.84, even if its susceptibility to sulfoxaflor was restored. Compared with the susceptible strain (Sus), the Sul-R and Sul-D strains became more active in searching for appropriate feeding positions because they generated more intercellular apoplastic stylar pathway events (C). However, the phloem-feeding ability was reduced in the Sul-R and Sul-D strains, as shown by the decrease in phloem behavioral parameters, such as phloem salivations (E1), phloem ingestion (E2), and the percentages of E1 and E2. The negative hormesis effect of sulfoxaflor on phloem feeding was observed in susceptible strain but not in Sul-R and Sul-D, as evidenced by the significant decreases in the number of E1, the duration of E1 and E2, and the percentage of E1 and E2 in the Sus strain. Sulfoxaflor resistance was unstable in *A*. *gossypii*, and there was still a fitness cost to *A*. *gossypii* after recovering susceptibility to sulfoxaflor. The phloem-feeding ability was reduced in the Sul-R and Sul-D strains compared with the Sus strain, but the negative hormesis effect of sulfoxaflor on phloem feeding was only found in the Sus strain. The outcomes of this study could contribute to a comprehensive risk assessment and provide a basis for developing a better strategy to control *A*. *gossypii*.

## Simple Summary

Cotton aphids are becoming resistant to sulfoxaflor and other insecticides, which is a big problem for cotton production in China. The resistance in the cotton aphis to sulfoxaflor is not permanent. After 22 generations without pressure, the resistant strain became susceptible to other insecticides. The resistant strain was more active but less able to feed on the phloem. Additionally, the negative effect of sulfoxaflor on phloem feeding was absent in the resistant strains. These findings highlight the need for a thorough risk assessment and improved strategies to manage the cotton aphid.

## 1. Introduction

The cotton aphid, *Aphis gossypii*, is a devastating cotton pest with resistance to a variety of insecticides, including pyrethroids, carbamates, organophosphates, and neonicotinoids [1,2]. In recent years, sulfoxaflor, classified as Group 4C by the Insecticide Resistance Action Committee [3], has been widely utilized to control cotton aphids in China, and it has been an important insecticide for IPM programs registered in 92 countries in the world to control many sucking insects [4]. Moreover, because sulfoxaflor has a special action mechanism that is different from other neonicotinoid insecticides, it has been extensively used to control resistant sucking insect pests, such as cotton aphids [5,6,7]. However, with excessive application of sulfoxaflor in the field as an essential alternative insecticide, some populations in different regions of *A. gossypii* have developed a high level of resistance to it [8,9]. By reducing the use of insecticides, rational rotation of different types of insecticides, and other comprehensive control measures, the development of resistance can be delayed [10]. However, after the application of insecticides is reduced, whether the sensibility of insects can return to close to the original level and all biological parameters in declined resistant strain can be restored to the same level as the susceptible strain has been unknown till now.

The biological and physiological characteristics, such as fitness and behavioral responses of *A. gossypii*, have changed while they had resistance to sulfoxaflor [11]. With insecticide selection pressure, the pest can evolve resistance to insecticides. Normally, pests respond to this pressure at the expense of fitness costs, as evidenced by high energy costs or a significant disadvantage in survival [12]. This decreased fitness has been reported in sulfoxaflor-resistant, thiamethoxam-resistant, clothianidin-resistant, and acetamiprid-resistant strains of *A. gossypii* [13,14,15,16]. The fitness costs associated with insecticide resistance can result in resistant populations being less competitive than susceptible populations, and pests can regain susceptibility to insecticides without insecticide pressure [17]. After resistance declines, the chlorpyrifos-resistant *Phenacoccus solenopsis* [18], sulfoxaflor-resistant *Nilaparvata lugens* [19], and acetamiprid-resistant *A. gossypii* [20] regain susceptibility to the screened insecticides, as well as to other insecticides to which they have previously been cross-resistant. However, it remains unclear whether this fitness cost associated with the resistance disappears after *A. gossypii* regains susceptibility to insecticides along with losing the selective pressure in the resistant strain.

The electrical penetration graph (EPG) has been widely used to study resistance characteristics in host plants, transmission mechanisms of plant pathogens, and modes of action of insecticides [21,22,23]. Insecticides may have an effect on aphid feeding behavior, and many studies have investigated the effects of insecticides on the feeding behavior of *Myzus persicae* [22] and *A. gossypii* [24,25,26] through EPG in recent years. Using this method, a previous report suggested that the sulfoxaflor-resistant *A. gossypii* were more active in finding an appropriate site for feeding than the susceptible aphids [24]. The sulfoxaflor could provoke more rapid and effective phloem feeding in the susceptible and sulfoxaflor-resistant aphids [25]. However, little is known about the aphid feeding behavior after its resistance declines.

In order to explore the fitness, cross-resistance, and feeding behavior changes in cotton aphids after their resistance level declined to sulfoxaflor, a series of experiments were conducted in this study. First, the stability of resistance in cotton aphids to sulfoxaflor was investigated. Second, the life table parameters of aphids in the sulfoxaflor-resistant and resistance-decline strains were compared with the susceptible strain by the age-stage, two-sex life table theory [27]. Finally, the feeding behaviors of three strains were analyzed by EPGs. The results of this study will contribute to understanding the risk assessment of resistance to sulfoxaflor in cotton aphids and provide a reference to the rational optimization of sulfoxaflor in the control strategy for cotton aphids.

## 2. Materials and Methods

### 2.1. Insect

The *A. gossypii* susceptible strain (Sus) was collected in 2019 from cotton in Jinghe County (82°90′ E, 44°59′ N), Xinjiang, and then reared in ZM49 cotton without exposure to any insecticides. The sulfoxaflor-resistant strain (Sul-R) was the same as that in our previous study [11], which exhibited a 51.57-fold resistance level compared to the Sus strain after successive resistance selection. The sulfoxaflor resistance-decline strain (Sul-D) was obtained from the Sul-R strain by rearing for 22 generations without exposure to any insecticide. All strains were kept in a greenhouse at 26 ± 5 °C, 70 ± 10% RH, and 16:8 L/D.

### 2.2. Insecticides and Reagents

Sulfoxaflor (95%) was obtained from Corteva Agriscience (Indianapolis, CA, USA). Acetamiprid (97% *w*/*w*) and imidacloprid were obtained from Jiangsu Weier Chemical Co., Ltd. (Yancheng, China). Triton X-100 was obtained from Beijing Coolaber Technology Co., Ltd. (Beijing, China). All other chemicals and solvents were analytical-grade reagents obtained from Sinopharm Chemical Reagent Co., Ltd. (Shanghai, China).

### 2.3. Bioassays

The toxicity of sulfoxaflor against the cotton aphid was determined using the leaf dipping method [28]. Briefly, stock solutions of insecticide were prepared in acetone and diluted in a series of five to six concentrations with 0.05% (*v*/*v*) Triton X-100. The 23 mm diameter cotton leaf discs were cut from fresh, insecticide-free cotton leaves using a sharpened steel punch and immersed for 15 s in the desired concentration of sulfoxaflor or 0.05% (*v*/*v*) Triton X-100 (as a control), and then air-dried in the shade. The dried cotton leaf discs were placed upside down onto 12-well cell culture plates containing agar medium (1%, 2 mL). The 30 apterous adult aphids were carefully transferred onto each cotton leaf disc and then covered with rice paper to prevent them from escaping. For each treatment, three replicates were established, with the mortality being evaluated after 48 h. All culture plates containing cotton aphids were placed in an artificial climate chamber at 26 ± 1 °C, 70 ± 5% RH, and 16 h:8 h L/D.

### 2.4. Establishment of Sulfoxaflor Resistance-Decline Strain

The initial Sul-D strain (G_0_) was derived from the Sul-R strain and reared independently without insecticide pressure. The bioassay was conducted on the Sul-D strain at intervals of one generation until its susceptibility to sulfoxaflor approached that of the Sus strain.

The decrease in resistance rate (DRR) was performed using the following formula:

$$DRR=(1-\frac{{LC}_{50}\;of\;G_{n}}{{LC}_{50}\;of\;G_{0}})\times 100\%$$

where G_0_ represented the initial Sul-D strain and G_n_ represented the subsequent declining generations.

The decrease in resistance (DR) was performed using the following formula [29]:

$$DR=\frac{\left[\mathrm{lg}\left(finalL\;C_{50}\right)-\mathrm{lg}(initial\;LC_{50})\right]}{N}$$

where N represented the number of generations without insecticide exposure.

### 2.5. Fitness Comparison of Three A. gossypii Strains

Life tables for the three strains in this study were tested following the methodology described by Ma et al. [14]. To obtain cotton aphids of the same age, about 500 adults from each strain were transferred to fresh cotton seedlings for oviposition. After 24 h, 100 first instar nymphs were randomly selected from each strain. Each nymph was placed on a 23 mm diameter disk of fresh cotton leaves, which were placed upside down in a 12-well cell culture plate containing 1% agar medium and covered with nylon mesh. Fresh cotton leaf discs were replaced every four days. The developmental time of the different stages and fecundity of cotton aphids were recorded daily until the death of the cotton aphids. The offspring were counted and removed every day. All cotton aphids were maintained in an artificial climatic chamber at 26 ± 1 °C, 70 ± 5% HR, and 16 h:8 h L/D.

### 2.6. Probing and Feeding Behavior Test

EPG signals of cotton aphid feeding behavior were monitored using a Giga-8 DC-EPG device (EPG-systems Wageningen, The Netherlands) and converted by an analog/digital (A/D) converter card (Di 710, EPG-systems, Wageningen, The Netherlands). The Giga-8 DC-EPG device includes insect electrodes and ground electrodes. For the insect electrode, one end of a gold wire (2 cm length × 18 μm in diameter) was attached to a copper wire utilizing water-soluble silver conductive paint and linked to the EPG electrode. In the same way, the other end of the copper wire was attached to the dorsum of the cotton aphid, which was placed on the abaxial surface of the cotton leaf to feed. For the ground electrode, a copper electrode (10 cm long × 2 mm diameter) was inserted into the soil near the cotton stem. The Giga-8 DC-EPG device was placed in a Faraday cage (50 cm long × 40 cm width × 50 cm high) in controlled conditions of 26 ± 1 °C and 70 ± 5% HR.

The experiment was set up with two treatments: (i) the Sus, Sul-R, and Sul-D strains fed solely on control plants treated with 0.05% Triton X-100 for EPG and (ii) the three strains fed on sulfoxaflor-treated plants treated with 0.898 mg·L^−1^ sulfoxaflor (LC_25_ value on Sus strain) for the EPG. All cotton aphids were starved for 2 h prior to testing [30]. Per treatment, 15–17 cotton aphids were recorded, and each individual specimen was continuously monitored for 8 h. Each replicate was used with new aphids and plants.

According to Rauch et al. [31], waveforms associated with aphid feeding behavior have been classified into the following categories: (i) waveform Np represents non-detecting periodic waveform; (ii) waveform C represents the intercellular apoplastic stylet pathway; (iii) waveform Pd represents probing inside living cells; (iv) waveform E1 represents salivation into phloem sieve elements; and (v) waveform E2 represents passive phloem sap uptake from the sieve element. The waveforms E1 and E2 were correlated with phloem activity.

### 2.7. Statistical Analyses

The LC_50_ values and 95% confidence intervals (95%CI) were estimated by probit analysis using SPSS 27.0 (SPSS Inc., Chicago, IL, USA). The resistance ratio (RR) was calculated by dividing the LC_50_ value of the Sul-R or Sul-D strain by the LC_50_ value of the Sus strain. Individual replicates were utilized as independent variables in each concentration gradient. LC_50_ value was deemed significantly distinct if their 95% confidence interval did not overlap [32].

Based on the age-stage, two-sex life table theory, the TWOSEX-MS Chart program was used to evaluate life table data for *A. gossypii* individuals. Using 100,000 random resampling in the TWOSEX-MS Chart [33], the bootstrap procedure was used to determine the means and standard errors (SE) of the parameters. The paired bootstrap test was utilized to compare variations in the parameters based on the confidence interval of the differences [34]. Relative fitness (*R_f_*) was determined by dividing the *R*_0_ value of the resistant strain by the *R*_0_ value of the field strain [14].

The number of waveform events per insect (NWEI) was calculated by dividing the sum of the number of events for a given waveform by the sum of the total number of insects in each treatment. The waveform duration per insect (WDI) was calculated by dividing the durations of each event of a given waveform produced by each individual insect producing that waveform by the total number of insects under each treatment [22]. The differences among treatments were analyzed by one-way ANOVA or Student’s *t*-test in SPSS 27.0 (SPSS Inc., Chiago, IL, USA).

## 3. Results

### 3.1. Resistance Declining to Sulfoxaflor in A. gossypii

The declining trend of resistance to sulfoxaflor in the Sul-D strain is shown in Table 1. After 22 generations without any insecticide pressure, the resistance in aphids to sulfoxaflor decreased in the Sul-D strain from 51.57 to 1.11-fold with a DR of −0.08. The decrease in resistance rate in the Sul-D strain reached more than 50% at the 8th generation and 96.53% at the 20th generation. The sensitivity of the Sul-D strain to sulfoxaflor was close to that of the Sus strain at the 22nd generation.

### 3.2. Cross-Resistance in Sul-R and Sul-D Strains

Cross-resistance in aphids in the Sul-R and Sul-D strains to acetamiprid and imidacloprid is shown in Table 2. The Sul-R strain showed apparent cross-resistance to acetamiprid (19.61-fold) and imidacloprid (53.58-fold) compared to the Sus strain (95% CIs nonoverlap). After 22 generations with no insecticide pressure, the susceptibility of aphids in the Sul-D strain to acetamiprid and imidacloprid was not significantly different from that in the Sus strain (95% CIs overlap).

### 3.3. The Fitness Change in the Sul-R and Sul-D Strains

#### 3.3.1. A Comparison of Fitness Between the Sus and Sul-R Strains

**Figure 1:**
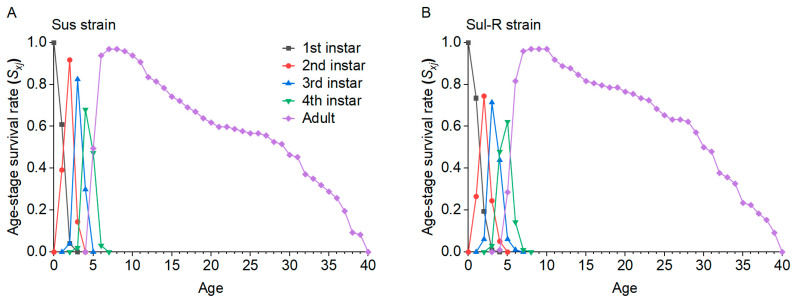
Age-stage-specific survival rates (*S_xj_*) in the Sus (**A**) and Sul-R (**B**) strains.

**Figure 2:**
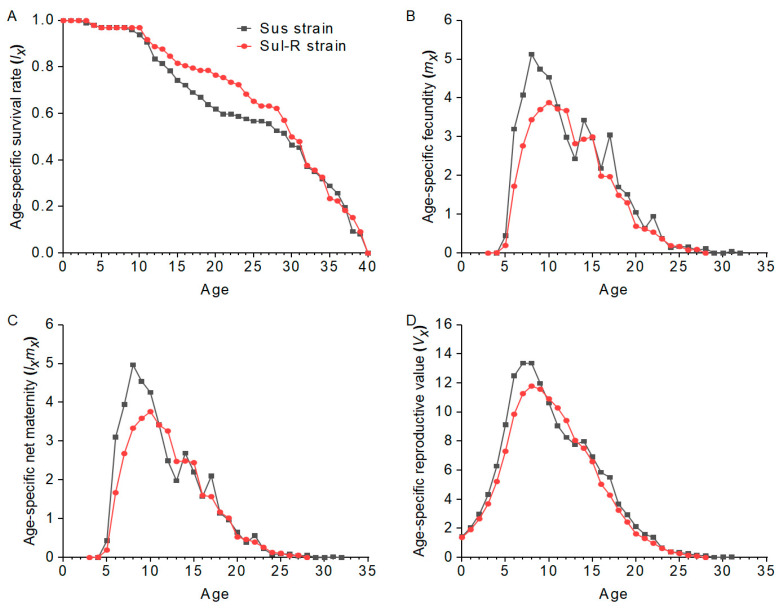
The age-specific survival rates (**A**), age-specific fecundity (**B**), age-specific net maternity (**C**), and age-specific reproductive value (**D**) in the Sus and Sul-R strains.

The biological and demographic parameters of the Sus and Sul-R strains are shown in Table 3. The developmental duration of the first instar nymph, pre-adult, and total preoviposition period (TPOP) in the Sul-R strain was significantly longer than that in the Sus strain. The developmental duration of the second instar nymph and fecundity of the Sul-R strain were significantly lower than those of the Sus strain. Compared to the Sus strain, the intrinsic rate of increase (*r*), finite rate of increase (*λ*), net reproductive (*R*_0_), and gross reproductive rate (GRR) of the Sul-R strain were significantly reduced, but its mean generation time (*T*) was significantly increased. The overall fitness of the Sul-R strain was 0.87 compared to the Sus strain. The age-stage survival rate (*S_xj_*) curves of nymph stages in the Sul-R strain were lower than those in the Sus strain, and the pattern of *S_xj_* curves of adults in the Sul-R strain was higher than that in the Sus strain (Figure 1). The age-specific survival rate (*lx*) curve in the Sul-R strain was higher than that in the Sus strain, especially at the ages of 11–32 days (Figure 2). The age-specific fecundity (*m_x_*) curves, age-specific net maternity (*l_x_m_x_*) curves, and age-specific reproductive value (*V_x_*) curves in the Sul-R strain were lower than those in the Sus strain (Figure 2). These results showed that the Sul-R strain of *A*. *gossypii* had a significant fitness cost.

#### 3.3.2. A Comparison of the Fitness Between the Sus and Sul-D Strains

**Figure 3:**
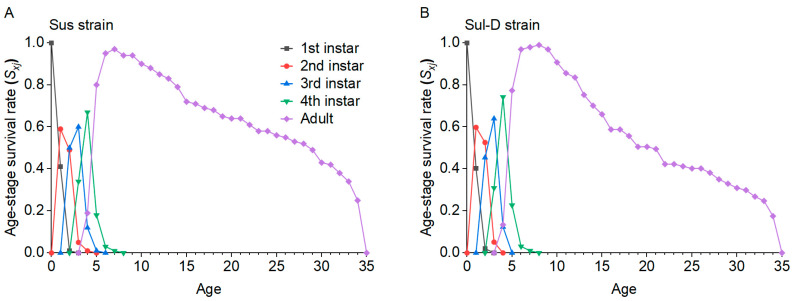
The age-stage-specific survival rates (*S_xj_*) in the Sus (**A**) and Sul-D (**B**) strains.

**Figure 4:**
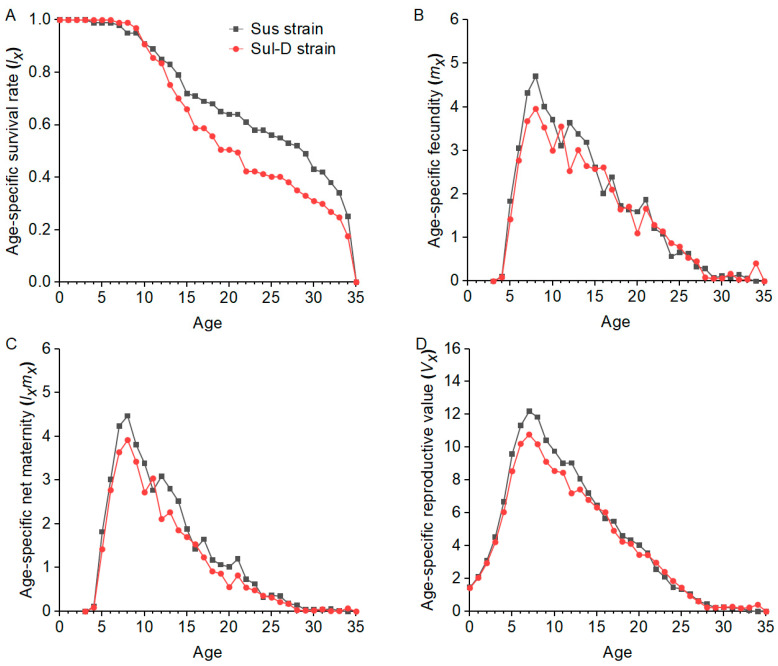
The age-specific survival rates (**A**), age-specific fecundity (**B**), age-specific net maternity (**C**), and age-specific reproductive value (**D**) in the Sus and Sul-D strains.

The biological and demographic parameters for the Sus and Sul-D strains are shown in Table 4. Compared to the Sus strain, significant reductions in adult longevity, total longevity, oviposition days, and fecundity were observed in the Sul-D strain. The *r*, *λ*, *R*_0_, and GRR of the Sul-D strain were significantly reduced compared to the Sus strain. The overall fitness of the Sul-D strain was 0.84 compared to the Sus strain. The pattern of *S_xj_* curves of adults in the Sul-D strain was lower than that in the Sus strain (Figure 3). The *lx*, *m_x_*, *l_x_m_x_*, and *V_x_* curves in the Sul-D strain were lower than those in the Sus strain (Figure 4). This indicated that the Sul-D also had a fitness cost compared with the Sus strain.

### 3.4. The Changes of Probing and Feeding Behavior in the Sul-R and Sul-D Strains Compared with the Sus Strain

Differences in the probing and feeding behavior were observed in the Sus, Sul-R, and Sul-D strains fed on control cotton plants treated with 0.05% triton X-100 (Table 5). Compared to the Sus strain, the Sul-R strain had a significantly lower number of non-probing (Np) waveforms (*p* = 0.047), the duration of phloem salivation (E1) (*p* = 0.003), and the percentage of E1 and E2 (*p* = 0.001). Similarly, the Sul-D strain had a significantly lower duration of E1 (*p* = 0.003), phloem ingestion (E2) (*p* = 0.017), and the percentage of E1 and E2 (*p* = 0.001), while having a significantly higher duration of Np (*p* = 0.008), compared to the Sus strain. Additionally, the number of E1 (*p* = 0.000) and E2 (*p* = 0.026) in the Sul-D strain were significantly lower than those in the Sul-R strain, but the duration of Np in the Sul-D strain was significantly higher than that in the Sul-R strain (*p* = 0.008). No significant differences were observed in other variables among the three strains.

### 3.5. The Sublethal Effects of Sulfoxaflor on the Probing and Feeding Behavior in the Sus, Sul-R, and Sul-D Strains

#### 3.5.1. The Sublethal Effects of Sulfoxaflor on the Probing and Feeding Behavior in the Sus Strain

The 0.898 mg·L^−1^ concentration (LC_25_ value on Sus strain) of sulfoxaflor markedly affected the probing and feeding behavior in the Sus strain (Table 6). Compared with control plants, the number of E1, the duration of E1 and E2, and the percentage of E1 and E2 were significantly lower in Sus strain aphids exposed to sulfoxaflor-treated plants. However, the number and duration of C and the time from the 1st C to 1st E2 in Sus strain aphids exposed to sulfoxaflor-treated plants were significantly higher than those exposed to control plants.

#### 3.5.2. The Sublethal Effects of Sulfoxaflor on the Probing and Feeding Behavior in the Sul-R Strain

The probing and feeding behavior in the Sul-R strain was less affected by the 0.898 mg·L^−1^ concentration (LC_25_ value in the Sus strain) of sulfoxaflor (Table 7), there was no significant difference in the variables between the two treatments.

#### 3.5.3. The Sublethal Effects of Sulfoxaflor on the Probing and Feeding Behavior in the Sul-D Strain

The probing and feeding behavior in the Sul-D strain under different treatments are shown in Table 8. The number of C and Pd and the duration of C and Pd in the Sul-D strain aphids exposed to sulfoxaflor-treated plants were significantly lower than those in the Sul-D strain aphids exposed to control plants. There were no significant differences in other variables.

## 4. Discussion

Chemical control is a crucial aspect of cotton pest management strategy, aimed at mitigating the negative impact of pest damage on cotton yield. This has led to an over-reliance on insecticides for cotton pest control, resulting in high levels of insecticide resistance in cotton pests, particularly cotton aphids [1,2,9]. Sulfoxaflor, the first sulfoximine insecticide [5], has been widely used for stinging pest control in recent years because it has high efficacy against resistant pests, which have high levels of resistance to many chemical insecticides. But while providing good control, field populations of different pests have developed varying degrees of resistance to sulfoxaflor due to non-scientific use of this insecticide [8,35,36]. Discontinuation or rotation of using insecticide can delay the development of pest resistance, and the physiological and behavioral response in the pest after the resistance level is reduced is critical to whether this insecticide can continue to be used. Therefore, in this study, we tested the changes in cross-resistance, fitness, and feeding behavior in *A. gossypii,* along with the decline in resistance to sulfoxaflor. The results will contribute to providing the reference to rational optimize sulfoxaflor in control strategy against cotton aphids.

Understanding the stability of resistance in pests after lost exposure selection to insecticides is of great practical importance. When pest resistance to an insecticide is unstable, then eliminating this insecticide from the spray program can reduce the pest resistance and thus prolong the effectiveness of this insecticide [37]. Unstable insecticide resistance has also been reported for chlorpyrifos-resistant *P. solenopsis* [18], acetamiprid-resistant *Bemisia tabaci* [37], *P. solenopsis* [38], and *A*. *gossypii* [20]. In this study, sulfoxaflor resistance was unstable, the resistance ratio in *A*. *gossypii* reduced from 51.57 to 1.11-fold with a DR of −0.08 after 22 generations without any insecticide pressure. Moreover, as sulfoxaflor resistance declined, cross-resistance in the Sul-D strain to acetamiprid and imidacloprid also declined.

Insects cope with different survival environments with degrees of fitness. To deal with insecticides, insects use a variety of behavioral, physiological, and genetic strategies, such as mutations in the target sites or overexpression of detoxification enzymes, resulting in costly consequences that may affect insect reproduction, dispersal ability, and fitness [12], Fitness costs associated with insecticide resistance have been reported in thiamethoxam-resistant *A*. *gossypii* [16], pymetrozine-resistant *N*. *lugens* [17], and methoxyfenozide-resistant *Musca domestica* [39]. Moreover, fitness cost varies due to the difference in pest populations and insecticide types [11]. The current study has shown that the sulfoxaflor-resistant strains of pests also have fitness costs, such as *M*. *persicae* [40] and *N*. *lugens* [41]. Our present study showed that the Sul-R strain of *A*. *gossypii* had a significant decrease in fecundity, *r*, *λ, R*_0_, *T*, and GRR, and its relative fitness declined to 0.87 compared with susceptible aphids. This result is similar to the findings of Ma et al. [14] and our previous study [11]. In addition, the susceptibility in the Sul-D strain to sulfoxaflor had recovered close to that in Sus strains after 22 generations without insecticide pressure. However, the relative fitness of the Sul-D strain was not recovered and still had a decreased fitness of 0.84, with a significant decrease in the adult longevity, total longevity, fecundity, *r*, *λ, R*_0_, and GRR. This indicates that there may still be a fitness cost in resistant cotton aphid populations even if they recover susceptibility to insecticides, and this is beneficial for the implementation of resistance management strategies by reducing insecticide use or insecticide rotation.

Nowadays, the electrical penetration graph (EPG) technique is widely used to explore the differences in probing and feeding behavior in sucking pests. Previous findings suggest that insecticide pressure affects aphid probing and feeding behavior [22,24,26,42]. Insects often require multiple explorations to find suitable feeding sites [43]. Wang et al. showed that the imidacloprid-resistant and sulfoxaflor-resistant *A. gossypii* were more active in searching for suitable feeding sites before their stylets arrived at the phloem, as evidenced by an increase in the number and duration of C waveform [24,26]. Similar results were also observed in the present study. In this study, the Sus, Sul-R, and Sul-D strains showed significant differences in probing and feeding behaviors when aphids fed on common plants. The number and duration of the C waveform were increased in both Sul-R and Sul-D strains despite no significant difference from the Sus strain. Moreover, the number of the Np waveform was reduced in both Sul-R and Sul-D strains. These results indicate that the Sul-R and Sul-D strains not only actively look for suitable feeding sites but also probe more quickly. However, the Sul-R and Sul-D strains had less ability in phloem feeding. They ingested from the phloem (time from the 1st C to 1st, and E1 and E2) more slowly than the Sus strain. Furthermore, parameters for the E1 and E2 waveform associated with phloem activity decreased in the Sul-R and Sul-D strains. For example, the number and duration of E1 and E2 were significantly decreased in the Sul-D strain, the duration of E1 was significantly decreased in the Sul-R strain compared with the Sus strain, and the percentage of E1 and E2 was significantly decreased in the Sul-R and Sul-D strains. The phloem sap is a vital source of energy for insects as it contains amino acids, saccharides, and various nutrients, which are crucial for insect growth and development [44]. The reduced ability of the Sul-R and Sul-D strains to feed makes them less efficient at obtaining energy from the phloem sap, which may lead to a reduction in their fitness.

The sublethal effects of insecticides on development, sex ratio, reproductive capacity, lifespan, feeding behavior, oviposition behavior, chemical communication, and other aspects of insects are known [45]. A previous study found that the hormesis effects of sulfoxaflor on phloem feeding are observed in susceptible and sulfoxaflor-resistant *A. gossypii*, with a significant increase in the duration and percentage of E2 [24]. However, in this study, negative sublethal effects of sulfoxaflor on phloem feeding were observed in the Sus strain. The number and duration of E1, the duration of E2, and the percentage of E1 and E2 were significantly reduced in the Sus strain when exposed to sulfoxaflor-treated plants (LC_25_ value on Sus strain). This result suggests that there is an uncertain effect of sulfoxaflor on the phloem-feeding of *A. gossypii*. This may be related to many factors affecting the sublethal effect of insecticides, such as insecticides, pest species, genetic backgrounds and generations of pests, and the use of sublethal treatment concentrations [45,46]. In addition, significant changes in phloem feeding were not observed in Sul-R and Sul-D strains under the same treatment. This indicates that this concentration of sulfoxaflor (LC_25_ value on Sus strain) could not affect the feeding in the Sul-R strain due to its high resistance to sulfoxaflor (51.57-fold). Previous studies have found that the P450 genes involving high resistance to sulfoxaflor are still overexpressed in sulfoxaflor resistance-decline *N. lugens* [19], which would result in an energy trade-off based on resource trade-offs theory [47,48]. In this study, the decrease in relative fitness of the Sul-D strain may be related to the fact that genes involved in sulfoxaflor resistance are still overexpressed in the Sul-D strain and that overexpression of such genes may have eliminated the effect of sulfoxaflor on Sul-D strain. Therefore, the expression of genes involved in sulfoxaflor resistance in the Sul-D strain needs to be investigated in the future.

## 5. Conclusions

Sulfoxaflor resistance was unstable in *A*. *gossypii*, which still had a decreased relative fitness even though it regained susceptibility to sulfoxaflor. The feeding ability of the Sul-R and Sul-D strains was reduced compared to that of the Sus strain. In addition, sulfoxaflor showed a negative effect on the phloem feeding in the Sus strain. The outcomes of this study will provide a basis for developing a better strategy to control *A*. *gossypii*.

## Figures and Tables

**Table 1:** The trend of resistance decline in the Sul-D strain of *Aphis gossypii*.

Generation	Slope ± SE ^a^	LC_50_ mg·L^−1^ 95%(CI) ^b^	*χ* ^2^	Resistance Ratio ^c^	Decrease in Resistance Rate (%) ^d^
Sus	1.47 ± 0.13	2.58 (1.85~3.45)	19.53	1	-
G_0_	1.59 ± 0.17	133.05 (107.95~164.98)	18.77	51.57	-
G_2_	1.05 ± 0.10	110.72 (80.28~158.02)	25.93	42.91	16.78
G_4_	1.39 ± 0.14	81.01 (63.64~101.49)	21.22	31.40	39.11
G_6_	0.92 ± 0.09	60.31 (44.30~82.05)	22.54	23.27	54.67
G_8_	0.95 ± 0.08	48.41 (36.37~64.44)	17.47	18.75	63.62
G_10_	0.74 ± 0.06	41.36 (29.28~58.78)	19.80	16.16	68.91
G_12_	2.27 ± 0.23	29.13 (22.68~35.63)	16.36	11.29	78.11
G_14_	0.98 ± 0.08	22.43 (16.49~29.82)	14.28	8.69	83.14
G_16_	1.24 ± 0.09	16.91 (12.73~21.83)	19.73	6.55	87.29
G_18_	2.07 ± 0.16	4.62 (3.76~5.69)	21.80	1.79	96.53
G_20_	1.05 ± 0.07	3.00 (2.15~4.05)	15.24	1.16	97.75
G_22_	1.11 ± 0.08	2.86 (2.02~3.90)	21.48	1.11	97.85

^a^ Standard error. ^b^ Confidence limits. ^c^ Resistance ratio = LC_50_ of G_n_/LC_50_ of Sus strain. ^d^ Decrease in resistance rate (%) = (1 − LC_50_ of G_n_/LC_50_ of G_0_) × 100%.

**Table 2:** Cross-resistance in Sul-R and Sul-D strains of *Aphis gossypii* to various insecticides.

Strain	Insecticide	Slope ± SE ^a^	LC_50_ mg·L^−1^ 95%(CI) ^b^	*χ* ^2^	Resistance Ratio ^c^
Sus	Sulfoxaflor	1.47 ± 0.13	2.58 (1.85~3.45)	19.53	1.0
	Acetamiprid	1.51 ± 0.18	4.32 (3.48~5.57)	10.22	1.0
	Imidacloprid	1.44 ± 0.20	5.91 (4.57~8.27)	5.81	1.0
Sul-R	Sulfoxaflor	1.59 ± 0.17	133.05 (107.95~164.98)	18.77	51.57
	Acetamiprid	2.31 ± 0.30	184.71 (155.97~219.52)	18.12	19.61
	Imidacloprid	1.07 ± 0.21	316.67 (221.15~590.12)	15.49	53.58
Sul-D	Sulfoxaflor	1.11 ± 0.08	2.86 (2.02~3.90)	21.48	1.11
	Acetamiprid	2.22 ± 0.28	5.93 (4.94~7.30)	7.78	1.37
	Imidacloprid	1.66 ± 0.29	9.92 (7.41~15.86)	3.24	1.68

^a^ Standard error. ^b^ Confidence limits. ^c^ Resistance ratio = LC_50_ in Sul-R or Sul-D strain/LC_50_ in Sus strain.

**Table 3:** The biological and demographic parameters for the Sus and Sul-R strains of *Aphis gossypii*.

Parameters ^a^	Sus Strain		Sul-R Strain		95% CL ^c^	*p*-Value
	n	Mean ± SE ^b^	n	Mean ± SE ^b^		
First instar nymph (d)	97	1.65 ± 0.06	98	1.94 ± 0.07	(0.11, 0.47) *	0.001
Second instar nymph (d)	95	1.44 ± 0.06	96	1.28 ± 0.05	(0.02, 0.30) *	0.025
Third instar nymph (d)	95	1.19 ± 0.04	95	1.31 ± 0.05	(−1.47, 0.25)	0.086
Forth instar nymph (d)	94	1.22 ± 0.04	95	1.33 ± 0.05	(−2.59, 0.23)	0.119
Pre-adult (d)	94	5.52 ± 0.06	95	5.86 ± 0.07	(0.16, 0.52) *	<0.001
APOP (d)	94	0.34 ± 0.06	95	0.47 ± 0.06	(−0.03, 0.29)	0.102
TPOP (d)	94	5.86 ± 0.06	95	6.34 ± 0.08	(0.29, 0.67) *	<0.001
Adult longevity (d)	94	21.32 ± 1.07	95	22.91 ± 0.92	(−1.18, 4.33)	2.261
Total longevity (d)	97	26.13 ± 1.10	98	28.02 ± 0.99	(−1.05, 4.80)	0.206
Oviposition days (d)	94	12.26 ± 0.48	95	12.54 ± 0.39	(−0.93, 1.49)	0.650
Fecundity (offspring/individual)	94	43.40 ± 1.62	95	37.91 ± 1.25	(1.55, 9.48) *	0.007
*r* (d^−1^)		0.36 ± 0.01		0.33 ± 0.01	(0.02, 0.05) *	<0.001
*λ* (d^−1^)		1.44 ± 0.01		1.39 ± 0.01	(3.35, 7.00) *	<0.001
*R*_0_ (offspring/individual)		42.06 ± 1.74		36.74 ± 1.37	(1.01, 9.66) *	0.016
*T* (d^−1^)		10.29 ± 0.12		11.03 ± 0.14	(0.39, 1.10) *	<0.001
GRR (d^−1^)		49.91 ± 1.37		41.42 ± 0.89	(5.37, 11.75) *	<0.001
R*_f_*		1		0.87		

^a^ APOP, adult pre-oviposition period; TPOP, total preoviposition period; *r*, intrinsic rate of increase; *λ*, finite rate of increase; *R*_0_, net reproductive rate; *T*, mean generation time; *GRR*, gross reproduction rate; *R_f_* = *R*_0_ in Sul-R strain/*R*_0_ of Sus strain. The ^b^ Mean ± SE was estimated using the bootstrap technique with 100,000 re-samplings. ^c^ An asterisk indicates a significant difference between the two strains at *p* < 0.05, paired bootstrap test using the TWOSEX MS chart program.

**Table 4:** Biological and demographic parameters for the Sus and Sul-D strains of *Aphis gossypii*.

Parameters ^a^	Sus Strain		Sul-D Strain		95% CL ^c^	*p*-Value
	n	Mean ± SE ^b^	n	Mean ± SE ^b^		
First instar nymph (d)	100	1.47 ± 0.08	97	1.42 ± 0.05	(−0.14, 0.25)	0.640
Second instar nymph (d)	100	1.14 ± 0.03	97	1.17 ± 0.04	(−0.06, 0.14)	0.491
Third instar nymph (d)	99	1.24 ± 0.04	97	1.21 ± 0.04	(−0.09, 0.14)	0.086
Forth instar nymph (d)	98	1.23 ± 0.04	97	1.32 ± 0.05	(−0.05, 0.22)	0.119
Pre-adult (d)	98	5.07 ± 0.10	97	5.13 ± 0.07	(−0.18, 0.29)	0.587
APOP (d)	98	0.39 ± 0.06	97	0.36 ± 0.06	(−0.13, 0.19)	0.750
TPOP (d)	98	5.50 ± 0.08	97	5.46 ± 0.11	(−0.23, 0.29)	0.790
Adult longevity (d)	98	20.42 ± 0.96	97	17.18 ± 0.97	(0.56, 5.90) *	0.018
Total longevity (d)	100	25.09 ± 0.96	97	22.32 ± 0.96	(0.10, 5.45) *	0.041
Oviposition days (d)	98	14.26 ± 0.63	97	12.29 ± 0.65	(0.21, 3.73) *	0.029
Fecundity (offspring/individual)	98	45.34 ± 2.08	97	37.22 ± 2.18	(2.19, 14.02) *	0.007
*r* (d^−1^)		0.38 ± 0.01		0.36 ± 0.01	(0.01, 0.03) *	0.031
*λ* (d^−1^)		1.46 ± 0.01		1.43 ± 0.01	(0.00, 0.05) *	0.034
*R*_0_ (offspring/individual)		44.42 ± 2.14		37.22 ± 2.18	(1.21, 13.15) *	0.018
*T* (d^−1^)		10.04 ± 0.11		10.03 ± 0.12	(−0.32, 0.33)	0.983
GRR (d^−1^)		54.26 ± 1.46		49.61 ± 1.72	(0.29, 9.11) *	0.039
R*_f_*		1		0.84		

^a^ APOP, adult pre-oviposition period; TPOP, total preoviposition period; *r*, intrinsic rate of increase; *λ*, finite rate of increase; *R*_0_, net reproductive rate; *T*, mean generation time; *GRR*, gross reproduction rate; *R_f_* = *R*_0_ in the Sul-D strain/*R*_0_ of Sus strain. The ^b^ Mean ± SE was estimated using the bootstrap technique with 100,000 re-samplings. ^c^ An asterisk indicates a significant difference between the two strains at *p* < 0.05, paired bootstrap test using the TWOSEX MS chart program.

**Table 5:** The probing and feeding behavior in the Sus, Sul-R, and Sul-D strains of *Aphis gossypii*.

Variables	Sus Strain		Sul-R Strain		Sul-D Strain	
	PPW ^a^	Mean ± SE	PPW ^a^	Mean ± SE	PPW ^a^	Mean ± SE
Number of waveform events per insect (NWEI)						
Np	15/15	14.33 ± 2.42 a	17/17	7.24 ± 0.87 b	16/16	11.81 ± 2.45 ab
C	15/15	167.27 ± 17.81 a	17/17	226.88 ± 20.14 a	16/16	202.25 ± 24.63 a
Pd	15/15	236.53 ± 17.51 a	17/17	211.88 ± 19.07 a	16/16	183.25 ± 22.69 a
E1	15/15	17.60 ± 1.44 a	17/17	14.00 ± 1.37 a	16/16	8.31 ± 1.16 b
E2	15/15	8.13 ± 1.45 a	17/17	8.65 ± 0.87 a	16/16	4.88 ± 0.69 b
Waveform duration per insect (WDI) (min)						
Np	15/15	27.52 ± 4.39 b	17/17	22.80 ± 6.01 b	16/16	49.03 ± 7.32 a
C	15/15	171.15 ± 11.74 a	17/17	193.32 ± 17.40 a	16/16	208.06 ± 21.93 a
Pd	15/15	16.00 ± 1.12 a	17/17	13.97 ± 1.22 a	16/16	12.29 ± 1.61 a
E1	15/15	91.86 ± 14.44 a	17/17	51.51 ± 6.93 b	16/16	37.98 ± 10.44 b
E2	15/15	208.34 ± 20.16 a	17/17	147.24 ± 21.66 ab	16/16	110.88 ± 26.20 b
Time from the 1st C to 1st Pd	15/15	0.09 ± 0.01 a	17/17	0.10 ± 0.01 a	16/16	0.11 ± 0.01 a
Time from the 1st C to 1st E1	15/15	70.87 ± 17.88 a	17/17	75.90 ± 11.51 a	16/16	108.84 ± 18.73 a
Time from the 1st C to 1st E2	15/15	124.19 ± 25.43 a	17/17	163.48 ± 21.95 a	16/16	148.85 ± 26.70 a
Other variables						
Percentage of E1 + E2	15/15	0.62 ± 0.04 a	17/17	0.42 ± 0.05 b	16/16	0.31 ± 0.06 b

^a^ PPW, the proportion of individuals that produced the waveform type. The different letters in the same row represent significant differences among the three strains at *p* < 0.05 by one-way ANOVA.

**Table 6:** Probing and feeding behavior in Sus strain of *Aphis gossypii* after treatment by sulfoxaflor.

Variables	Aphid Fed on Control Plants		Aphid Fed on Sulfoxaflor-Treated Plants		*p* Value
	PPW ^a^	Mean ± SE	PPW ^a^	Mean ± SE	
Number of waveform events per insect (NWEI)					
Np	15/15	14.33 ± 2.42 a	15/15	10.13 ± 1.81a	0.176
C	15/15	167.27 ± 17.81 b	15/15	234.40 ± 25.06 a	0.038
Pd	15/15	236.53 ± 17.51 a	15/15	215.07 ± 23.29 a	0.467
E1	15/15	17.60 ± 1.44 a	15/15	9.87 ± 1.66 b	0.002
E2	15/15	8.13 ± 1.45 a	15/15	5.80 ± 0.97 a	0.192
Waveform duration per insect (WDI) (min)					
Np	15/15	27.52 ± 4.39 a	15/15	38.13 ± 7.36 a	0.226
C	15/15	171.15 ± 11.74 b	15/15	245.56 ± 16.97 a	0.001
Pd	15/15	16.00 ± 1.12 a	15/15	14.40 ± 1.36 a	0.370
E1	15/15	91.86 ± 14.44 a	15/15	43.67 ± 7.67 b	0.006
E2	15/15	208.34 ± 20.16 a	15/15	110.42 ± 25.86 b	0.006
Time from the 1st C to 1st Pd	15/15	0.09 ± 0.01 a	15/15	0.12 ± 0.02 a	0.183
Time from the 1st C to 1st E1	15/15	70.87 ± 17.88 a	15/15	104.09 ± 20.39 a	0.231
Time from the 1st C to 1st E2	15/15	124.19 ± 25.43 b	15/15	215.80 ± 32.22 a	0.034
Other variables					
Percentage of E1 + E2	15/15	0.62 ± 0.04 a	15/15	0.28 ± 0.05 b	0.000

^a^ PPW, the proportion of individuals that produced the waveform type. The different letters in the same row represent significant differences at *p* < 0.05 by Student’s *t*-test.

**Table 7:** The probing and feeding behavior in the Sul-R strain of *Aphis gossypii* after being treated by sulfoxaflor.

Variables	Aphid Fed on Control Plants		Aphid Fed on Sulfoxaflor-Treated Plants		*p* Value
	PPW ^a^	Mean ± SE	PPW ^a^	Mean ± SE	
Number of waveform events per insect (NWEI)					
Np	17/17	7.24 ± 0.87 a	15/15	8.93 ± 1.17 a	0.247
C	17/17	226.88 ± 20.14 a	15/15	239.07 ± 24.40 a	0.700
Pd	17/17	211.88 ± 19.07 a	15/15	219.93 ± 22.50 a	0.785
E1	17/17	14.00 ± 1.37 a	15/15	11.33 ± 2.00 a	0.271
E2	17/17	8.65 ± 0.87 a	15/15	5.87 ± 1.24 a	0.072
Waveform duration per insect (WDI) (min)					
Np	17/17	22.80 ± 6.01 a	15/15	29.22 ± 9.90 a	0.574
C	17/17	193.32 ± 17.40 a	15/15	214.41 ± 23.68 a	0.472
Pd	17/17	13.97 ± 1.22 a	15/15	14.58 ± 1.34 a	0.736
E1	17/17	51.51 ± 6.93 a	15/15	42.3 ± 11.12 a	0.464
E2	17/17	147.24 ± 21.66 a	15/15	124.51 ± 31.14 a	0.546
Time from the 1st C to 1st Pd	17/17	0.10 ± 0.01 a	15/15	0.09 ± 0.01a	0.683
Time from the 1st C to 1st E1	17/17	75.90 ± 11.51 a	15/15	104.09 ± 20.39 a	0.224
Time from the 1st C to 1st E2	17/17	163.48 ± 21.95 a	15/15	194.02 ± 33.76 a	0.444
Other variables					
Percentage of E1 + E2	17/17	0.42 ± 0.05 a	15/15	0.35 ± 0.06 a	0.385

^a^ PPW, the proportion of individuals that produced the waveform type. The different letters in the same row represent significant differences at *p* < 0.05 by Student’s *t*-test.

**Table 8:** The probing and feeding behavior in the Sul-D strain of *Aphis gossypii* after being treated by sulfoxaflor.

Variables	Aphid Fed on Control Plants		Aphid Fed on Sulfoxaflor- Treated Plants		*p* Value
	PPW ^a^	Mean ± SE	PPW ^a^	Mean ± SE	
Number of waveform events per insect (NWEI)					
Np	16/16	11.81 ± 2.45 a	15/15	7.73 ± 1.56 a	0.177
C	16/16	202.25 ± 24.63 a	15/15	136.40 ± 18.79 b	0.044
Pd	16/16	183.25 ± 22.69 a	15/15	123.13 ± 17.25 b	0.046
E1	16/16	8.31 ± 1.16 a	15/15	6.33 ± 1.15 a	0.238
E2	16/16	4.88 ± 0.69 a	14/15	4.29 ± 0.75 a	0.567
Waveform duration per insect (WDI) (min)					
Np	16/16	49.03 ± 7.32 a	15/15	83.46 ± 25.00 a	0.204
C	16/16	208.06 ± 21.93 a	15/15	164.52 ± 22.09 a	0.173
Pd	16/16	12.29 ± 1.61 a	15/15	8.24 ± 1.07 b	0.047
E1	16/16	37.98 ± 10.44 a	15/15	49.18 ± 15.20 a	0.544
E2	16/16	110.88 ± 26.20 a	15/15	130.21 ± 29.93 a	0.629
Time from the 1st C to 1st Pd	16/16	0.11 ± 0.01 a	15/15	0.10 ± 0.01 a	0.560
Time from the 1st C to 1st E1	16/16	108.84 ± 18.73 a	15/15	178.00 ± 39.59 a	0.130
Time from the 1st C to 1st E2	16/16	148.85 ± 26.70 a	14/15	234.02 ± 43.06 a	0.107
Other variables					
Percentage of E1 + E2	16/16	0.31 ± 0.06 a	15/15	0.36 ± 0.06 a	0.605

^a^ PPW, the proportion of individuals that produced the waveform type. The different letters in the same row represent significant differences at *p* < 0.05 by Student’s *t*-test.

## Data Availability

The data presented in this study are available on request from the corresponding authors.
